# Books and Authors

**Published:** 1909-09

**Authors:** 


### BOOKS AND AUTHORS.

**Modern Medicine.** Its Theory and Practice. In Original Contributions by American and Foreign Authors. Edited by **William Osler,** M.D., Regius Professor of Medicine in Oxford University, England. Assisted by Thomas McCrea, M.D., Associate Professor of Medicine and Clinical Therapeutics in Johns Hopkins University. In seven octavo volumes, illustrated. Volume VI., Diseases of the Urinary System, of the Ductless Glands, of the Muscles, Diseases of Obscure Causation, Vasomotor and Trophic Disorders, Medical Aspects of Life Insurance. Lea & Febiger, Publishers, Philadelphia and New York, 1909. (Price per volume: cloth, $6.00; leather, $7.00; half morocco, $7.50 net prices.)

This volume is one of special interest covering, as it does, a very wide and important range of subjects—namely, the diseases of the urinary system, of the ductless glands, of the muscles, those of obscure causation, vasomotor and trophic disorders, and the medical aspects of life insurance. These diseases are all handled by specially competent men. John McCrae, of Toronto, begins the volume with two chapters on the kidney, followed by two on urinary anomalies and uremia by Garrod, of London. Herrick, of Chicago, deals with all aspects of nephritis, as well as amyloid disease, and Thomas R. Brown, of Baltimore, considers pyogenic and tubercular affections of the kidney. Its medico-surgical aspects, from the pen of H. H. Young, of Baltimore, conclude this section. George Dock, formerly of Ann Arbor, now of New Orleans, has written the entire section on the ductless glands. Longcope, of Philadelphia, considers Hodgkin's Disease; T. McCrae, of Baltimore, arthritis deformans; Dock, of New Orleans, osteomalacia; and D. J. McCarthy, of Philadelphia, astasia-abasia and adiposis dolorosa. Together with W. R. Steiner, of Hartford, McCarthy has written the section on muscular diseases. The editor, Dr. Osler, with his former colleague, C. P. Emerson, of Baltimore, handles the section on vasomotor and trophic disorders, and Charles Lyman Greene, of St. Paul, concludes with the medical aspects of life insurance.

This brief statement of the contents gives a fairly good idea of the value of the book. The authors of the articles are among the best known clinicians of the English-speaking world of medicine. The standard of this treatise is a high one but it is maintained through every volume of the series.

---

**Saunders's Question Compends.** Essentials of Medical Chemistry. By **Lawrence Wolff.** Seventh edition, revised by A. Ferree Witmer. Philadelphia and London: W. B. Saunders Co. (Price, $1.00.)

Twenty years have elapsed since this compend first appeared. Besides the seven editions that have been issued it has been reprinted a number of times, hence its usefulness has the approval of those who need it in no uncertain manner. Such a book on chemistry is valuable as a reminder to essentials that are more or less difficult to retain. There is scant need of extended remarks relating to a book so well known. Suffice it to say that it is one of the best of its class.

---

**Dietetics for Nurses.** By **Julius Friedenwald,** Professor of Gastroenterology in the College of Physicians and Surgeons, Baltimore, and **John Ruhrah,** Professor of Diseases of Children in the College of Physicians and Surgeons, Baltimore. Second edition. 12mo, pp. 395. Philadelphia and London: W. B. Saunders Co. (Cloth, $1.50.)

When this book appeared first, four years ago, it assumed at once a place of importance in its appropriate class. Indeed, it may be said almost to form a class by itself. Dietetics for nurses is important because nurses are in constant relation to the sick, hence a nurse must know, of a fact, how food should be prepared for patients suffering from any disease. This book serves as a reminder in any emergency, and is standard authority on the subject of which it treats. Nurses everywhere need the information given here, which is not only of the very best, but is plainly told, and cannot fail to do good whenever its instructions are followed. The milk article has been revised and a chapter has been added, giving a number of simple methods relating to detection of food adulterations and preservatives. A

number of other changes have been made in the book bringing its essentials up to the last moment.

---

**Hydrotherapy.** By **William H. Dieffenbach,** Professor of Hydrotherapy, New York Homeopathic Medical College and Flower Hospital. Octavo, pp. 267. Illustrated. New York: Rebman Co. (Cloth, $3.00.)

This author has mastered the art of hydrotherapy and has put his knowledge into book form in an interesting manner. There is scarcely a region or an organ of the body to which water, hot or cold, warm or freezing, is not applied either directly or indirectly in the management, or treatment of disease, according to this treatise. It is full of descriptive methods for baths of all sorts, as well as the local application of water at all temperatures, —hot packs, hot bags, hot coils, ice bags, and every conceivable contrivance for the external use of water. Nor has the internal employment of water been neglected. It is well understood by educated people as well as by physicians that water is a valuable agent in the treatment of many diseases. All this, too, is herein delineated with skilful knowledge, making the book a complete exponent of hydrotherapy and a guide to appropriate methods.

---

**American Pocket Dictionary.** Edited by **W. A. Newman Dorland,** Assistant Obstetrician to University Pennsylvania Hospital. Sixth edition. Philadelphia and London: W. B. Saunders Co. (Flexible leather, $1.25.)

The popularity of this pocket dictionary is attested by the fact that six editions of it have appeared in ten years. This edition has been revised thoroughly and several hundred new words have been added, making it more complete than ever. In these days when so many new terms spring up in medicine at such short intervals, a small dictionary like this becomes a veritable necessity. One must keep it on the office desk for daily reference, when it is inconvenient or unnecessary to consult a larger work. This is one of the best of its kind and deserves its popularity.

